# Combination of antiplatelet and anticoagulant therapy, component network meta-analysis of randomized controlled trials

**DOI:** 10.3389/fcvm.2022.1036609

**Published:** 2022-12-08

**Authors:** László Szapáry, Dániel Tornyos, Péter Kupó, Réka Lukács, Oumaima El Alaoui El Abdallaoui, András Komócsi

**Affiliations:** Department of Interventional Cardiology, Heart Institute, Medical School, University of Pécs, Pécs, Hungary

**Keywords:** anticoagulant therapy, antiplatelet, meta-analysis, percutaneous coronary intervention (PCI), combination (combined) therapy

## Abstract

**Background:**

Despite numerous randomized clinical trials (RCT), data regarding the efficacy of antiplatelet and anticoagulant combinations are still conflicting. We aimed to analyze treatment options tested in various fields of cardiovascular prevention, regarding their efficacy and bleeding risk.

**Methods:**

Systematic searches of electronic databases were conducted until June 2022. A component network meta-analysis was performed in R. Risk estimates across trials were pooled using random-effects model selecting risk ratio (RR) with 95% confidence intervals (95% CIs) as summary statistics. The primary endpoint of interest was the rate of major cardiac adverse events (MACE). Major bleeding events were assessed as main safety endpoint. Secondary outcomes included cardiovascular- and overall mortality, myocardial infarction (MI), stent thrombosis, and stroke.

**Results:**

Fifteen studies randomizing 73,536 patients were identified. The MACE risk reflected heterogeneity among the anticoagulants with dabigatran and apixaban significantly reducing the risk of MACE (RR 0.56; 95% CI 0.39–0.80 and RR 0.75; 95% CI 0.58–0.98, respectively). Vitamin K antagonist (VKA), rivaroxaban, or edoxaban did not reduced of MACE while it was associated with a significant increase of bleeding risk (RR 1.66; 3.66, and 5.47, respectively). The direct anticoagulant (DOAC) dose reduction resulted in tendencies of fewer bleeding but higher MACE risk, while combination with aspirin was followed with increased risk for bleeding, however, remained non-significant in these cases.

**Conclusion:**

Our meta-analysis supports that the ischemic-bleeding balance is different among direct-acting oral anticoagulants (DOACs) while this is not significantly affected by the dose reduction approaches. Long-term aspirin treatment as part of the anticoagulant and dual antiplatelet regimen provides no ischemic benefit but may increase bleeding risk.

**Systematic review registration:**

[https://www.crd.york.ac.uk/prospero/], identifier [259703].

## Graphical Abstract

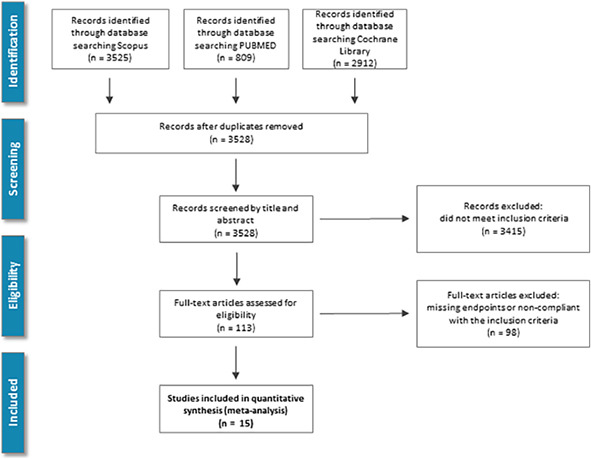

## Highlights

- When combined with antiplatelets, the safety and efficacy of different anticoagulants are still controversial. A network meta-analysis of 15 randomized controlled trials was performed.
- Analyzed in a network model that considers the individual elements of different combinations, anticoagulants show heterogeneous safety and efficacy.
- The direct anticoagulant (DOAC) dose reduction resulted in tendencies of lower bleeding but higher MACE risk, however, these effects remained non-significant in both cases.
- Long-term ASA provides no ischemic benefit but may increase bleeding risk.

## Introduction

One of the most difficult tasks for a 21st-century cardiologist is to find a balance between ischemia and bleeding complications (1). In the last decade, direct-acting oral anticoagulants (DOACs) have been introduced and have gradually displaced Vitamin-K antagonists (VKA) from the treatment in multiple indications including stroke prevention of non-valvular atrial fibrillation and treatment and prevention of deep vein thrombosis (2). Moreover, several trials showed promising results with DOACs or DOAC containing combinations in patients with a high risk of arterial events (3). The combination of drugs with different mechanisms of action may improve their potential in reducing the risk of ischemic events. However, the consequent higher bleeding risk may undermine this benefit (4). Antiplatelet and anticoagulant combinations were tested in various fields of cardiovascular risk prevention including cases with or without recent ischemic events or coronary intervention (5). Also, due to the high prevalence of significant coronary heart disease among patients with atrial fibrillation (AF) multiple studies were performed in this area (6, 7).

However, from the data of these trials, it is hard to abstract the benefits and risk profile of a specific agent. Moreover, these studies and their meta-analyses did not answer some clinically relevant questions. These include whether there is a disparate effect, among the direct anticoagulant (DOAC) agents when used as part of the antithrombotic combinations and if the effects between reduced and full therapeutic doses of DOACs verifies dose reduction in this context. Furthermore, with regards to the elements of antiplatelet therapy, the risk-benefit balance of aspirin is not fully elucidated. As it has been illustrated in the COMPASS (8) and the AUGUSTUS (9) trials, both bleeding and ischemic events show additive features which support the use of component network meta-analysis (CNMA) in the context of combined antiplatelet and anticoagulant regimes.

Therefore, we performed a systematic review with multiple treatment network meta-analysis (NMA) to balance the differences between the antiplatelet and anticoagulant combinations treatments. We aimed to compare the safety and efficacy and to analyze the risk of ischemic and hemorrhagic events attributable to the individual elements. For this aim the NMA supplemented with a CNMA modeling was used.

## Methods

### Literature search and data extraction

This systematic review was performed according to the standards of the PRISMA Extension Statement for Reporting of Systematic Reviews Incorporating Network Meta-analyses of Healthcare Interventions (10) and it is registered with PROSPERO (International Prospective Register of Systematic Reviews).

The authors collected data from three online databases: Medline (PubMed), Cochrane Library, and Scopus until 01 July 2022 from articles reporting randomized clinical trials (RCT) with combinations of DOACs and antiplatelet therapy. No language restriction was used.

The search strategy included terms related to DOACS (“rivaroxaban,” “BAY 59-7939,” “dabigatran,” “apixaban,” “edoxaban,” “DU-176b,” “betrixaban,” “PRT054021,” and “PRT064445”) in different combinations restricting the query to RCT.

Removal of duplicates was performed in a reference manager software (EndNote X7.5 Thomson Reuters, NY, USA). Studies were included if they fulfilled the following criteria: (a) randomized controlled trials published after 1 January 2000, (b) assessing the clinical safety and/or efficacy of a combined antiplatelet and anticoagulant regime, (c) reported event from a minimum follow-up duration of 30 days. Articles that met pre-defined eligibility criteria were chosen for full-text screening and were reviewed by the two investigators against criteria as outlined in the PICO framework as “in patients at substantial risk for acute cardiac or cerebrovascular events including patients with a history of cerebrovascular, coronary, or peripheral artery disease (P), whether an intervention with combined antiplatelet and anticoagulants (I) compared to placebo or different antithrombotic combination (C) has a favorable effect on prognostically relevant outcomes defined as major cardiovascular adverse events (MACE) and major bleeding (O).” Two investigators (LS and DT) independently evaluated records; any discrepancies were resolved by a third investigator (AK).

Studies were excluded if any of the following criteria applied: (a) non-randomized studies, (b) single-arm studies, (c) outcomes of interest were not reported or were impossible to extract or calculate from published results, or (d) duplicate publications.

The selected full-text articles entered the data extraction. Abstracted information included the following: first author, year of publication, study name, the applied doses of antithrombotic drugs, total numbers of patients, follow-up duration, primary- and secondary endpoints, protocol definitions of bleeding, as well as patient and procedural characteristics including mean age, sex, the following risk factors: AF, acute coronary syndrome (ACS), chronic coronary syndrome, coronary heart disease, and rate of percutaneous coronary intervention at admission.

### Endpoint definitions

The primary efficacy outcome of our analysis was the occurrence of MACE as defined by the composite of cardiovascular mortality, myocardial infarction (MI), and stroke. Major bleeding events were assessed as main safety endpoints. In case of the availability of multiple major bleeding definitions, we extracted The Thrombolysis in Myocardial Infarction (TIMI) major bleeding if available. Secondary outcomes included cardiovascular and overall mortality, MI, stent thrombosis, and stroke. As safety outcomes frequency of minor and the major and minor bleeding complications were also evaluated. For definitions of ischemic events, the internal definitions of the included trials were used. The data from intention to treat analyses were extracted and the endpoints of interest were collected until the longest follow-up available.

The full- and reduced-dose DOAC groups described in the results were grouped based on the dosage, determined by the U.S. Food and Drug Administration (FDA), for the purpose of stroke prevention.

The methodological qualities of the studies were also assessed using the Cochrane Collaboration tool for assessing the quality of RCTs.

### Network meta-analysis modeling

Considering that the trials used different arms for comparing outcomes of different antiplatelet and anticoagulant schemes including combinations as well as monotherapy of various antithrombotics we pre-specified the use of multiple treatment NMA supplemented with CNMA modeling. Calculations were performed in the R statistical software package version 4.0.3 [R Development Core Team (11, 12)] using the packages “meta 4.15-1” and “netmeta 1.2-1.” A *p*-value < 0.05 was considered to represent statistical significance.

Each potential combination was entered first as an individual study arm, and data were pooled in a multiple treatment NMA that allows for multiple direct and indirect intervention comparisons to be integrated into the analysis. We imputed the relevant treatment effect as risk ratio (RR) and its standard error and used a frequentist approach to construct a computational NMA model accounting for the correlating treatment effects. Within this model, nodes were defined as the individual study arms and combined effect estimates with their 95% confidence interval (CI) were then calculated for each edge combined in a random-effect network.

Values of *I*^2^ representing the amount of inconsistency and Cochran’s Q statistics and its corresponding *p*-value measuring the heterogeneity in the network were also calculated. *I*^2^ values < 25% indicated a low degree of heterogeneity; *I*^2^ > 25% but <50% indicated moderate heterogeneity; and *I*^2^ > 50% indicated substantial heterogeneity (13).

A special case encountered in our network was that treatment arms may be combinations of other treatments or have common components. Therefore, the influence of individual components was intended to be evaluated in an additive model assuming that the effect of treatment combinations is the sum of the effects of its components. For CNMA a model implementing an additive model function was used (14, 15).

Estimates for all treatment combinations are presented as league tables separating the pooled effect sizes of the direct comparisons and the NMA effect sizes for each comparison. For easier interpretation effect sizes are depicted in forms of forest plots with warfarin-based triple therapy set as reference. Furthermore, comparative ranking of the treatments according to the *P*-scores method assuming a treatment to be of higher rank if the rate of events is lower was also performed. The *P*-score ranking system is a frequentist analog of SUCRA (SUrface Under the Cumulative Ranking curve) that measures the certainty that one treatment is better than another treatment, averaged over all competing treatments (14).

Consistency analyses assess that the direct evidence in a network for the effect size between two treatments does not differ from the indirect evidence calculated for that same comparison. The assumption of consistency was assessed by net-heat plots as well as by net-splitting. The latter method splits our network estimates into the contribution of direct and indirect evidence, which allows us to control for inconsistency in specific comparisons (15).

To assess publication bias, a comparison-adjusted funnel plot, (an extension of the common funnel plot in cases of multiple treatment comparisons) was used displaying Eggers’ test results in support (16).

## Results

**FIGURE 1:**
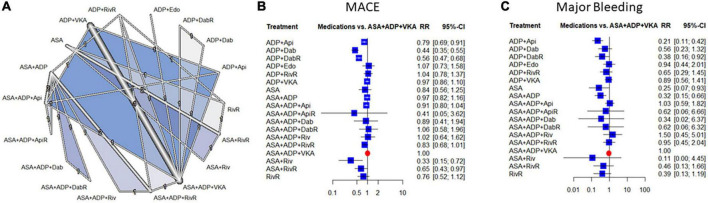
Evidence network **(A)**, and combination-level analyses of the relative risk (RR) of major adverse cardiovascular events (MACE) and major bleeding **(B,C)**. The forest plots depict the RR and their 95% confidence interval (CI) of the primary endpoints respective to the Vitamin K antagonist (VKA) and double antiplatelet therapy triple regime in the network meta-analysis (NMA). ASA, aspirin; ADP, P2Y12 ADP receptor antagonist; VKA, vitamin-K antagonist anticoagulation; Riv, rivaroxaban; Api, apixaban; Dab, dabigatran; Edo, edoxaban; RivR, reduced dose rivaroxaban; ApiR, reduced dose apixaban; DabR, reduced dose dabigatran.

A total of 15 RCTs met the selection criteria and contained sufficient data for statistical analysis, including a total of 73,536 patient data (Supplementary Figure 1). The different antithrombotic regimens were treated separately based on whether they contained a reduced or full dose of anticoagulant (Figure 1). These included six multiarm trials, including five studies with three arms and one trial with double randomization. The studies covered 14 protocols including seven anticoagulant + double antiplatelet, and eight anticoagulant + antiplatelet monotherapy combinations. Six trials included patients with AF with ACS and/or coronary stent implantation (5–7, 9, 17, 18) six studies randomized cases after an event of ACS (19–24), while in two trials antithrombotic combinations were tested in stable patients with a high risk of cardiovascular events (CVE) (8, 25) (Table 1 and Supplementary Table 1). The included studies were of high quality without major risk of bias (Supplementary Figure 2). The comparison-adjusted funnel plot showed no signs of important publication bias (Supplementary Figure 3). Neither net heat plots nor net-splitting analyses revealed major inconsistencies between direct and indirect evidence (Supplementary Figures 4, 5).

**TABLE 1:** Baseline clinical characteristics.

Study	References	Age years ± SD	Male gender no. (%)	AF (%)	CCS (%)	ACS (%)	STEMI (%)	NSTEMI (%)	Unstable angina (%)	Patient with CHD (%)	PCI (%)
ATLAS ACS-TIMI 46	Mega et al. (19)	58.1 (9.4)	2,690 (77.2)	0	0	100	52	30.6	17.4	0	64.2
APPRAISE-2	Alexander et al. (20)	67 (9.1)	5,014 (67.8)	0	0	100	39.6	41.6	18.1	0	44.6
ATLAS ACS 2-TIMI 51	Mega et al. (21)	61.7 (9.2)	11.600 (74.7)	0	0	100	50.3	25.6	24	0	60.4
REDEEM	Oldgren et al. (22)	61.8 (11.4)	1,414 (76)	0	0	100	60	40	0	0	54.5
APPRAISE-J	Ogawa et al. (23)	64.6 (9.5)	131 (86.8)	0	0	100	75,5	15.2	9,3	0	99.3
WOEST	Dewilde et al. (5)	69.9 (7.5)	448 (78.1)	69	45	55	NA	NA	NA	0	100
ISAR-TRIPLE	Fiedler et al. (17)	73.6 (8.2)	471 (76.7)	84	68	32.2	0,9	14.85	16.45	0	100
PIONEER	Gibson et al. (6)	70.1 (8.9)	4,069 (88.2)	100	47.8	52.2	12.4	18.6	21.2	0	100
COMPASS	Eikelboom et al. (8)	68.2 (7.9)	21.375 (78.0)	0	90.6	0	0	0	0	9.5	0
GEMINI-ACS-1	Ohman et al. (24)	62 (12.1)	2,275 (75)	0	0	100	49	40	11	0	87
RE-DUAL PCI	Cannon et al. (7)	70.2 (8.2)	2,664 (97.8)	100	41	48	NA	NA	NA	0	100
AFIRE	Yasuda et al. (25)	74.3 (8.3)	1,751 (78.3)	100	100	0	0	0	0	0	0
AUGUSTUS	Lopes et al. (26)	70.7 (13.2)	3,277 (71.0)	100	38.8	61.2	NA	NA	NA	0	64.2
ENTRUST-AF PCI	Vranckx et al. (18)	69.5 (14.1)	1,120 (75)	100	48	52	NA	NA	NA	0	100
MANJUSRI	Lu et al. (27)	69.4 (8.1)	188 (64)	100	228	64	NA	NA	NA	0	100

ACS, acute coronary syndrome; AF, atrial fibrillation; CHD, coronary heart disease; CCS, chronic coronary syndrome; NA, not applicable; NSTEMI, non-ST segment elevation myocardial infarction; PCI, percutaneous coronary intervention; STEMI, ST-segment elevation myocardial infarction.

### Combination-level analyses

Compared to the warfarin and dual-antiplatelet therapy the risk of MACE was significantly lower with rivaroxaban + aspirin, dabigatran + P2Y12 inhibitor and apixaban + P2Y12 inhibitor combinations [RR: 0.33 95% confidence interval (95% CI) (0.15–0.72), RR: 0.44; 95% CI: (0.35–0.55) and RR: 0.79; 95% CI: (0.69–0.91), respectively]. The risk reduction reached the level of statistical significance also with the reduced dose dabigatran [RR: 0.56; 95% CI: (0.47–0.68)]. There was a moderate degree of heterogeneity across the studies included in the analysis [*I*^2^: 39.8%, CI: (0.0%; 67.4%)] (Figure 1).

Overall mortality and cardiovascular mortality data did not show major differences, except for a significant mortality reduction with rivaroxaban + aspirin [RR: 0.31; 95% CI: (0.12–0.84)] (Supplementary Figure 6). Data reflected low levels heterogeneity with these outcomes [*I*^2^: 21.3%, CI: (0.0%; 63.5%), and 0%, CI: (0%; 68.3%)]. Compared to the VKA based triple therapy dual antiplatelet therapy + reduced dose rivaroxaban improved the risk of MI [RR: 0.66; 95% CI: (0.45–0.98)], ASA + reduced rivaroxaban improved the risk of stroke [RR: 0.36; 95% CI: (0.13–0.96)], and none of the combinations affected the risk of stent thrombosis (Supplementary Figure 7).

With regards to major bleeding two combinations of P2Y12 inhibitor with apixaban or with reduced-dose dabigatran decreased the risk significantly [RR: 0.21; 95% CI: (0.11–0.42), and RR: 0.38; 95% CI: (0.16–0.92)], respectively. There was a low degree of heterogeneity across the studies included in the analysis [*I*^2^: 35.5%, CI: (0.0%; 71.5%)]. The data of minor bleeding reflected similar trends, however, this difference reached only the level of statistical significance in the case of the apixaban-based dual therapy (Supplementary Figure 8).

**FIGURE 2:**
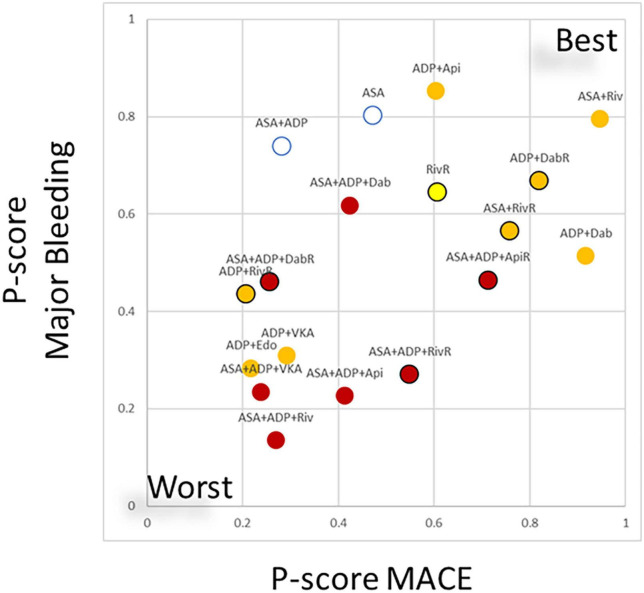
Clustering treatments based on ranking regarding the risk of major adverse cardiovascular events (MACE) and major bleeding risk. In the scatterplot the ranking (expressed as *P*-score values regarding the individual endpoints, ranging from 0–worse to 1–best) of treatment combinations are plotted. The values displayed a moderate correlation (*r* = 0.5) between the ischemic benefit and the bleeding risk associated to the different combinations. Combinations of anticoagulant therapies and double antiplatelet therapy are plotted as red, anticoagulant + single antiplatelet therapy is marked with orange anticoagulant monotherapy marked with yellow. Black margin of the marker marks regimes where reduced dose direct anticoagulant (DOAC) was applied. ASA, aspirin; ADP, P2Y12 ADP receptor antagonist; VKA, vitamin-K antagonist anticoagulation; Riv, rivaroxaban; Api, apixaban; Dab, dabigatran; Edo, edoxaban; RivR, reduced dose rivaroxaban; ApiR, reduced dose apixaban; DabR, reduced dose dabigatran.

Clustering treatments based on the treatment ranking regarding their ischemic and bleeding benefit showed a moderate correlation between these two characteristics (*r* = 0.50) (Figure 2).

### Component-level analyses

In the component-level analyses, the use of anticoagulants as part of the combinations resulted in a consistent increase of bleeding risk ranging from RR 2.35–5.47 (Subgroup heterogeneity: Cochrane chi^2^ *p* = 0.73, *I*^2^ = 0%). Except for apixaban and dabigatran, this effect was significant. The analysis reflected the higher rate escorted by edoxaban and VKA The effect of DOACs did not differ from VKA (*p* = 0.72).

Results with regards to the MACE risk reflected heterogeneity among DOACs (Subgroup heterogeneity: Cochrane chi^2^ *p* = 0.05, *I*^2^ = 63%). Dabigatran and apixaban significantly reduced the risk of MACE (54 and 25% RR reduction, respectively). While in the case of the other anticoagulants this effect remained non-significant with a trend of increase at edoxaban and VKA.

The supplementation of the treatment with ASA did not result in a reduction of MACE while it was associated with a non-significant trend of 66% increase in bleeding risk.

**FIGURE 3:**
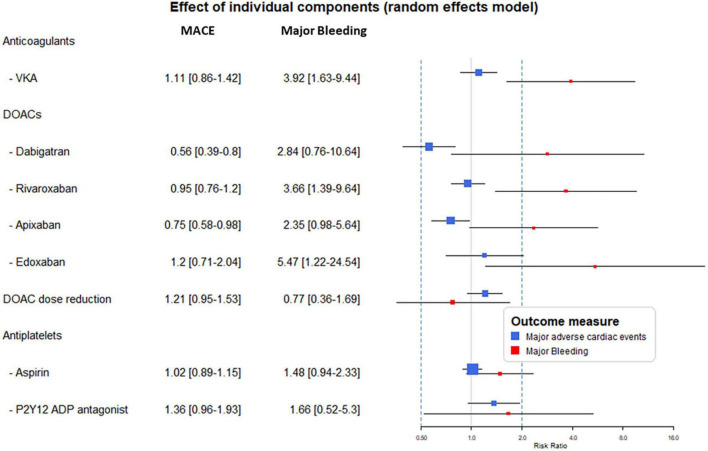
Results from the component network meta-analysis (CNMA) model analyses. The forest plot displays the risk ratio (RR) and 95% confidence interval (CI) attributable to the individual components when applied in an antithrombotic combination. MACE, major adverse cardiovascular events; VKA, vitamin-K antagonist; DOAC, direct oral anticoagulant; ADP, adenosine diphosphate.

The reduction of the DOAC dose showed tendencies of lower bleeding but higher MACE risk, however, remained non-significant in both cases (Figure 3). Subgroup analyses showed unvarying results supporting the consistency of the findings (Table 2).

**TABLE 2:** Results of the patient population stratified analyses.

A	Effect of individual components in the combinations [Relative risk (95% confidence interval)] major adverse cardiovascular events							

Subgroup model selection		VKA	Dabigatran	Rivaroxaban	Apixaban	Edoxaban	Reduction	Aspirin
Full model	14 trials 72,483 pts	1.11 (0.86–1.42)	0.56 (0.39–0.8)	0.75 (0.58–0.98)	0.95 (0.76–1.2)	1.2 (0.71–2.04)	1.21 (0.95–1.53)	1.02 (0.89–1.15)
AF studies (100%)	5 trials 13,205 pts	1.22 (1.04–1.42)	0.56 (0.46–0.68)	1.03 (0.71–1.5)	1.04 (0.87–1.25)	1.37 (0.97–1.93)	1.29 (0.99–1.67)	1.05 (0.94–1.17)
AF patients included	7 trials 14,392 pts	1.2 (1.02–1.42)	0.57 (0.45–0.71)	1.03 (0.7–1.52)	1.03 (0.84–1.26)	1.38 (0.96–1.98)	1.29 (0.97–1.71)	1.07 (0.95–1.21)
AF as exclusion criteria	7 trials 58,091 pts	NA	1 (0.53–1.89)	0.77 (0.56–1.07)	0.95 (0.79–1.14)	NA	1.06 (0.77–1.46)	0.86 (0.73–1)
ACS trial (100%)	6 trials 30,696 pts	NA	0.98 (0.51–1.9)	0.79 (0.55–1.13)	0.96 (0.73–1.26)	NA	1.09 (0.78–1.53)	0.82 (0.53–1.27)
ACS cases > 50%	9 trials 38,940 pts	1.03 (0.82–1.3)	0.97 (0.51–1.85)	0.83 (0.59–1.16)	0.91 (0.75–1.11)	1.1 (0.67–1.81)	1.1 (0.8–1.53)	1 (0.87–1.14)
ACS cases included	12 trials 42,852 pts	1.11 (0.86–1.43)	0.58 (0.4–0.82)	0.79 (0.6–1.04)	0.95 (0.76–1.2)	1.26 (0.74–2.14)	1.22 (0.96–1.54)	1.06 (0.92–1.23)
PCI trials (100%)	5 trials 7,542 pts	1.16 (0.76–1.75)	0.59 (0.38–0.91)	1.03 (0.57–1.85)	NA	1.43 (0.81–2.54)	1.29 (0.74–2.24)	1.16 (0.77–1.75)
Dominantly PCI trial (>50%)	11 trials 35,460 pts	1.13 (0.76–1.68)	0.59 (0.38–0.92)	0.79 (0.59–1.06)	0.98 (0.63–1.52)	1.29 (0.7–2.37)	1.22 (0.95–1.56)	1.06 (0.91–1.24)
Patients without coronary disease excluded	13 trials 45,088 pts	1.11 (0.86–1.43)	0.58 (0.4–0.82)	0.79 (0.6–1.04)	0.95 (0.76–1.2)	1.26 (0.74–2.14)	1.22 (0.96–1.54)	1.06 (0.92–1.23)
Phase 2 studies excluded	10 trials 64,705 pts	0.99 (0.77–1.28)	0.44 (0.29–0.66)	0.67 (0.47–0.95)	0.88 (0.7–1.1)	1.06 (0.63–1.79)	1.29 (0.93–1.77)	1 (0.89–1.14)

**B**		**Effect of individual components in the combinations [Relative risk (95% confidence interval)] major bleeding**						

**Subgroup model selection**		**VKA**	**Dabigatran**	**Rivaroxaban**	**Apixaban**	**Edoxaban**	**Reduction**	**Aspirin**

Full model		3.92 (1.63–9.44)	2.84 (0.76–10.64)	3.66 (1.39–9.64)	2.35 (0.98–5.64)	5.47 (1.22–24.54)	0.77 (0.36–1.69)	1.48 (0.94–2.33)
AF studies (100%)		1.01 (0.31–3.25)	0.94 (0.16–5.63)	1.24 (0.13–11.83)	0.54 (0.1–2.81)	1.59 (0.21–11.94)	0.68 (0.08–6.17)	1.67 (0.48–5.75)
AF patients included		1.13 (0.43–2.95)	0.83 (0.18–3.86)	1.23 (0.17–9.05)	0.61 (0.15–2.45)	1.41 (0.25–7.97)	0.68 (0.1–4.77)	1.33 (0.6–2.98)
AF as exclusion criteria		NA	2.04 (0.19–21.28)	3.34 (1.19–9.35)	2.7 (1.04–7.01)	NA	0.82 (0.34–1.99)	1.6 (0.76–3.37)
ACS trial (100%)		NA	2.15 (0.25–18.75)	5.11 (2.34–11.13)	2.6 (1.52–4.42)	NA	0.81 (0.42–1.55)	3.3 (1.17–9.28)
ACS cases > 50%		5.12 (1.28–20.57)	1.72 (0.11–27.07)	4.23 (0.92–19.51)	2.81 (0.77–10.25)	7.21 (0.67–77.73)	0.94 (0.27–3.22)	1.49 (0.62–3.57)
ACS cases included		4.49 (1.45–13.92)	2.98 (0.6–14.84)	4.51 (1.29–15.79)	2.58 (0.85–7.83)	6.27 (0.89–44.01)	0.81 (0.32–2.06)	1.48 (0.79–2.78)
PCI trials (100%)		1.04 (0.67–1.6)	0.72 (0.46–1.11)	1.11 (0.58–2.11)	NA	1.21 (0.76–1.93)	0.68 (0.33–1.39)	1.24 (0.8–1.93)
Dominantly PCI trial (>50%)		4.57 (1.12–18.68)	2.96 (0.51–17.19)	4.51 (1.19–17.07)	2.65 (0.52–13.62)	6.33 (0.72–55.53)	0.82 (0.31–2.16)	1.47 (0.76–2.85)
Patients without coronary disease excluded		4.49 (1.45–13.92)	2.98 (0.6–14.84)	4.51 (1.29–15.79)	2.58 (0.85–7.83)	6.27 (0.89–44.01)	0.81 (0.32–2.06)	1.48 (0.79–2.78)
Phase 2 studies excluded		3.82 (1.36–10.78)	2.99 (0.54–16.68)	3.88 (0.82–18.37)	2.23 (0.81–6.12)	5.07 (0.96–26.82)	0.68 (0.17–2.66)	1.41 (0.86–2.31)

The tables present the risk ratio (RR) and 95% confidence intervals (CIs) of the risk of major adverse cardiovascular events **(A)** and major bleeding **(B)** attributable to the individual components when applied in an antithrombotic combination. Stratification was done based on the main inclusion characteristics of the trials. VKA, vitamin K antagonist; AF, atrial fibrillation; ACS, acute coronary syndrome; PCI, percutaneous coronary intervention; NA, not available.

**FIGURE 4:**
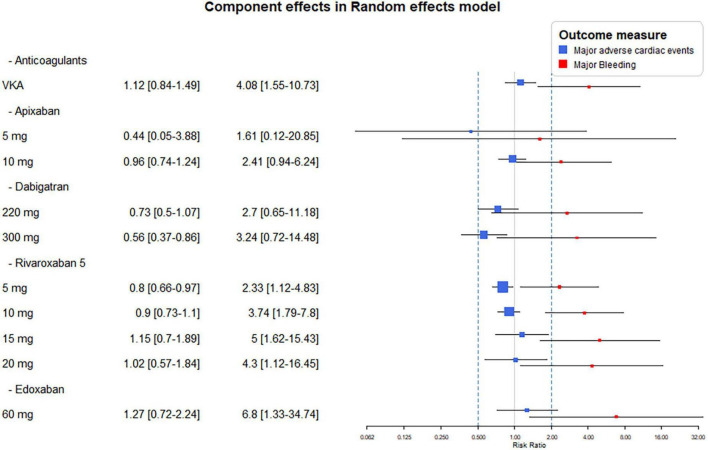
Combination-level analyses of the relative risk (RR) of major adverse cardiovascular events (MACE) and major bleeding. The forest plots depict the RR and their 95% confidence interval (CI) of the primary endpoints respective to the Vitamin K antagonist (VKA) and dose adjusted direct-acting oral anticoagulants (DOACs).

Component effect analyses of the different dose of DOACs showed similar effects of apixaban 5 or 10 mg daily with both major bleeding and the MACE endpoints. Bleeding risk were similar with both doses of dabigatran, however, significant reduction of MACE was seen only with its higher dose. The lowest dose of rivaroxaban (2,5 mg bid) reduced MACE significantly, while all dosing resulted in significant increase in bleeding risk (Figure 4).

## Discussion

The correct use of combined anticoagulant and antiplatelet therapy is still a challenge. Increasing use of coronary interventions, longer life expectancy, and comorbidities make it exceedingly difficult to find the right combination, dose, and treatment duration. To date, several studies have been conducted comparing different antithrombotic regimens, however, due to the high number of potential combinations these data represent a challenge for interpretation in daily practice.

Using a component NMA in a wide range of randomized trials testing combined antithrombotic medication we found a moderate correlation between the preventive efficacy and the ensuing bleeding risk. Data of the ischemic events showed major differences among the anticoagulants with dabigatran and apixaban being the most effective in this aspect while the increase of bleeding risk was homogenously featured with all anticoagulants. Dose reduction with DOAC based regimes did not significantly affect the outcome. The use of aspirin did not improve the rate of ischemic events.

Parallel to the increasing numbers of cardiovascular interventions important progress in the field of adjunctive pharmacotherapy can be observed. In recent years, several studies have compared different antithrombotic regimens. While preventive potential increases with intensification using more potent combined regimes the consequent higher risk of bleeding may offset these benefits (28).

Among aging patients and patients with multiple risk factors, there is an increasing proportion of patients with a need for chronic anticoagulation, most often due to AF. Many of these patients may undergo PCI with stent implantation, where dual antiplatelet treatment (DAPT) is recommended to avoid stent thrombosis. However, DAPT alone is inadequate to protect against the thromboembolic complication of AF (3). Thus, currently, this patient group represents the majority where combined antithrombotic therapy is applied.

In the 2010s, with the advent of DOACs showing better safety and similar efficacy profile as vitamin K antagonist (VKA), the clinical practice changed in terms of preventing thromboembolic complications in AF patients. Before the DOAC era, register analyses highlighted the potential hazards of the VKA based triple therapy, while the WOEST trial supported that withholding aspirin could improve outcomes (5, 29). The WOEST trial showed not only higher bleeding rates with a combination of DAPT plus warfarin compared with warfarin plus clopidogrel alone in patients receiving long-term OAC therapy and undergoing PCI but also a lower rate of ischemic events (5).

Recently, multiple studies were published comparing the use of different DOACs with VKAs in patients with non-valvular AF following PCI (6, 7, 9, 18). Dual antithrombotic therapy (DAT) including a DOAC and single antiplatelet treatment with a P2Y12 inhibitor improved safety outcomes reducing both total and major bleeding events significantly compared with TAT including warfarin plus DAPT in the PIONEER-AF (6) and RE-DUAL PCI (7) trials. The ENTRUST AF-PCI trial using edoxaban-based DAT was non-inferior to TAT and this trial did not find significant differences in the ischemic events (18). Nonetheless, these studies were underpowered to detect variations in terms of the efficacy of the DAT regimen versus triple therapy, however, both the treatment strategies were equally effective in terms of CVE rate, including MI, stroke and cardiac revascularization. Moreover, the design of these trials prevented the identification of risks specific to aspirin use and those associated with the different DOAC agents. In the AUGUSTUS trial AF patients undergoing PCI were randomized to receive apixaban or VKA and to receive aspirin or matching placebo for 6 months (9). Apixaban was associated with less bleeding and similar rates of CVEs compared with warfarin-based triple therapy regimen; however, numerically increased incidence of stent thrombosis and MI raised an important concern about DAT. Remarkably, the rate of ACS and particularly STEMI patients were limited (prevalence of ACS varied from 37.3 to 52%) in these trials where the ischemic risk is higher than in elective PCI population (9).

Earlier analyses including only two of the currently published four DOAC trials found a twofold higher risk of MI with DAT when compared with the TAT regimen (30, 31).

In 2019 Gargiulo et al. performed their meta-analysis including all the above four trials (32). They found that DAT is associated with a reduction of bleeding including major and intracranial hemorrhages–especially, but not exclusively if consisting of a DOAC and a P2Y12 inhibitor. Importantly, the bleeding benefit associated with DAT comes with a trade-off of cardiac but not cerebrovascular ischemic events. Notably, a recently published registry analysis of AMI patients receiving long-term OAC treatment found that patients who received aspirin had a lower risk of mortality and composite of cardiac events compared to those without aspirin (33).

The earlier network meta-analyses from these studies in line with our results supported that among the potential treatment options triple therapy should be avoided and the less intensified DOAC + P2Y12 inhibitor therapy offers a beneficial alternative mostly due to the reduction of bleeding risk (26). However, the fact that these analyses disregarded the medications used concomitant to the anticoagulation makes the interpretation of their results possible merely at the level of the combinations. With the help of a statistical methodology enabling the analysis of the risk associated with the components in the antithrombotic regimes, we found that the ischemic-bleeding balance is different with the different DOACs while this is not significantly affected by the dose reduction approaches applied in multiple trials.

In line with these earlier analyses, we found that the supplementation of the combined anticoagulant and antithrombotic combination with long-term aspirin do not improve the clinical outcome but increases the bleeding risk. It is of note that a recent analysis of the data from the AUGUSTUS trial showed that early aspirin cessation may also represent an excess of clinical risk and identified an optimal period of use of aspirin in the first week after the coronary intervention (34).

Another important aspect is that DOACs showed dissimilar results regarding cardiovascular safety. A meta-analysis of 28 randomized controlled trials involving 196,761 patients showed considerable heterogeneity among OACs (35). Head-to-head comparisons are not available regarding different DOACs in cases of AF patients undergoing PCI. A recently published NMA found that as part of the DAT apixaban was ranked first as the preferred therapy in terms of major or clinically relevant non-major bleeding and stroke, rivaroxaban ranked first as the preferred therapy in terms of MI and stent thrombosis, while dabigatran ranked first as the preferred therapy in terms of all-cause mortality (36). Various DOACs may have different risk-benefit profiles in combination strategies ensuring implementation of an optimal individualized antithrombotic regimen for patients receiving long-term OAC and undergoing PCI (35, 36).

With DOACs different dosing regimens may also influence the reached level of anticoagulation and interfere with the risk-benefit balance of the antithrombotic schemes. With this regard, our analysis found that although tendencies exist that with reduced dose DOAC schemes the bleeding risk decreases and it was counteracted by an increased risk of ischemic events, but none of this represented a significant effect.

In conclusion, this comprehensive analysis of randomized trials explored differences in risk-benefit balance among DOAC agents and rebuts the use of DOAC dose-reduction in the context of combined antithrombotic medication.

## Data availability statement

The original contributions presented in this study are included in the article/Supplementary material, further inquiries can be directed to the corresponding author.

## Author contributions

All authors listed above contributed to the final content of the publication and accepted all subsequent modifications and the final form of the article.
