# Supplementary material for: Combination of antiplatelet and anticoagulant therapy, component network meta-analysis of randomized controlled trials
Source: Front Cardiovasc Med. 2022 Dec 8;9:1036609. doi: 10.3389/fcvm.2022.1036609 (PMC9773199; doi:10.3389/fcvm.2022.1036609)
Supplement: Supplementary file 1 [file Data_Sheet_1.docx]

Supplemental material

Combination of antiplatelet and anticoagulant therapy component network meta-analysis of randomized controlled trials

László SZAPÁRY 1*, MD, Dániel TORNYOS1, MD, (0000-0003-1406-4016); Péter KUPÓ1, MD; Réka Lukács MD1, Oumaima EL ALAOUI EL ABDALLAOUI1, MSc; András KOMÓCSI1, MD, DSc, (0000-0002-8170-1778)*: contributed equally to the manuscript

Department of Interventional Cardiology, Heart Institute, Medical School, University of Pécs, Hungary

Contents

[Figure S1 Study screening and selection flow. 2](#_Toc115634566)

[Figure S2 Bias assessment 3](#_Toc115634567)

[Figure S3 Assessment of publication bias. 5](#_Toc115634568)

[Figure S4 Inconsistency heat plots of MACE outcome (Panel A) and major bleeding (Panel B) 6](#_Toc115634569)

[Figure S5. Net splitting plot of the major cardiovascular adverse events (MACE) (Panel A) and major bleeding (Panel B) showing the consistency between direct and indirect comparisons. 7](#_Toc115634570)

[Figure S6 Results of the network analysis of mortality outcomes. 8](#_Toc115634571)

[Figure S7 Results of the network analysis of ischemic outcomes. 9](#_Toc115634572)

[Figure S8 Results of the network analysis of minor, and major or minor bleeding events. 10](#_Toc115634573)

[Table S1 Baseline characteristics of the involved studies 11](#_Toc115634574)

# Figure S1 Study screening and selection flow.

Overview of study screening and selection process according to PRISMA guidelines

#####
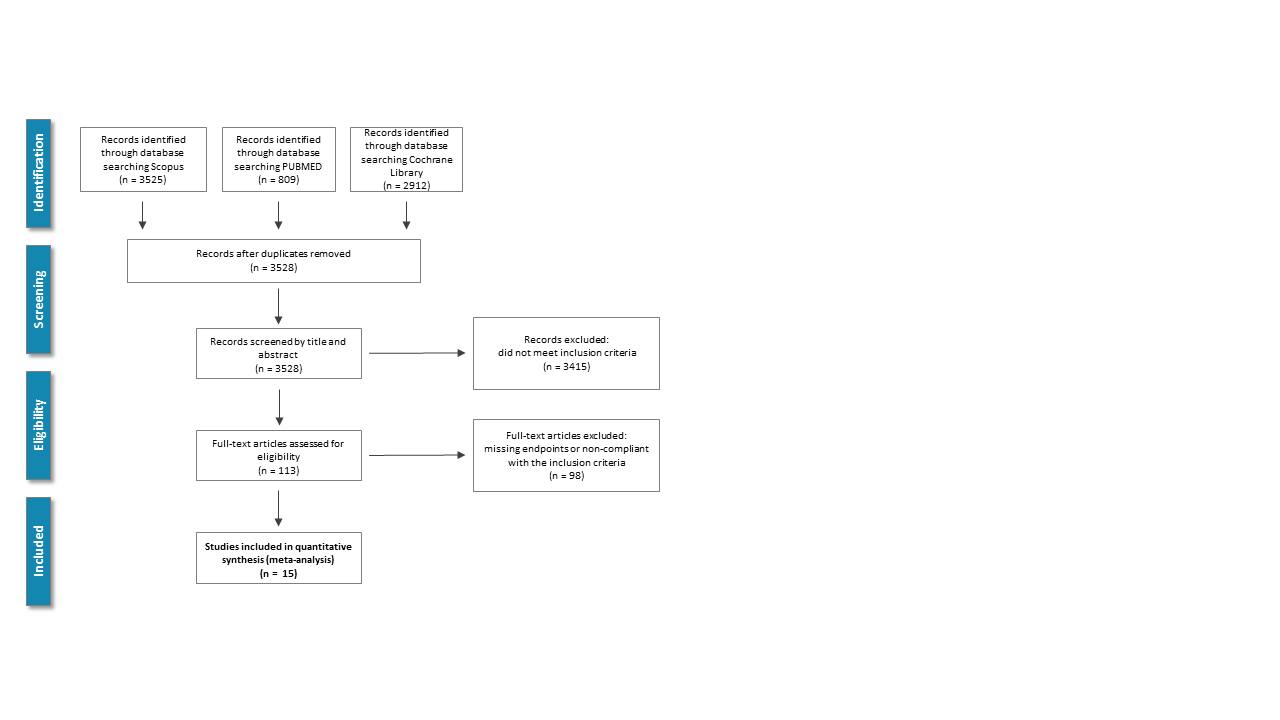


Figure S2 Bias assessment

The methodological quality of the included randomized control trials was assessed with the Cochrane Risk Bias tool.

2/A Bias assessment graph.


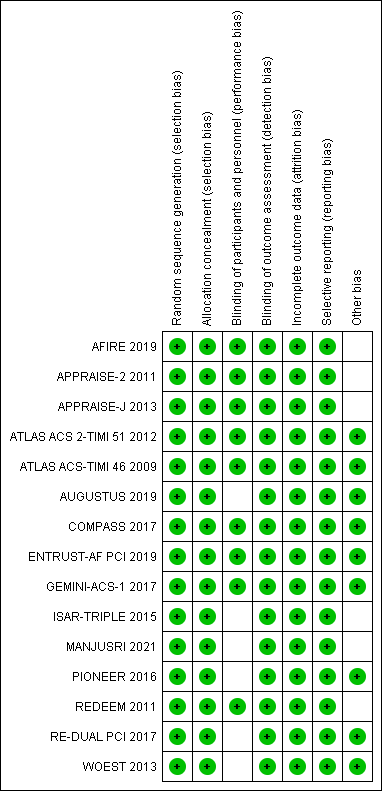


2/B Bias assessment summary report.


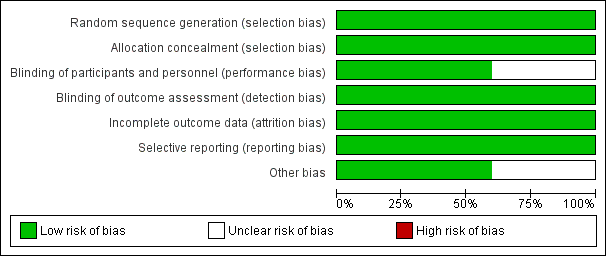


##### Figure S3 Assessment of publication bias. Comparison-adjusted funnel plot showed no signs of important publication bias. *Panel A*: major adverse cardiac events, *Panel B*: major bleeding *Abbreviations*: ASA: aspirin, ADP: P2Y12 ADP receptor antagonist, VKA: Vitamin-K antagonist anticoagulation, Riv: rivaroxaban, Api: apixaban, Dab: dabigatran, Edo: edoxaban, RivR: reduced dose rivaroxaban, ApiR: reduced dose apixaban, DabR: reduced dose dabigatran, vs: versus

##### A
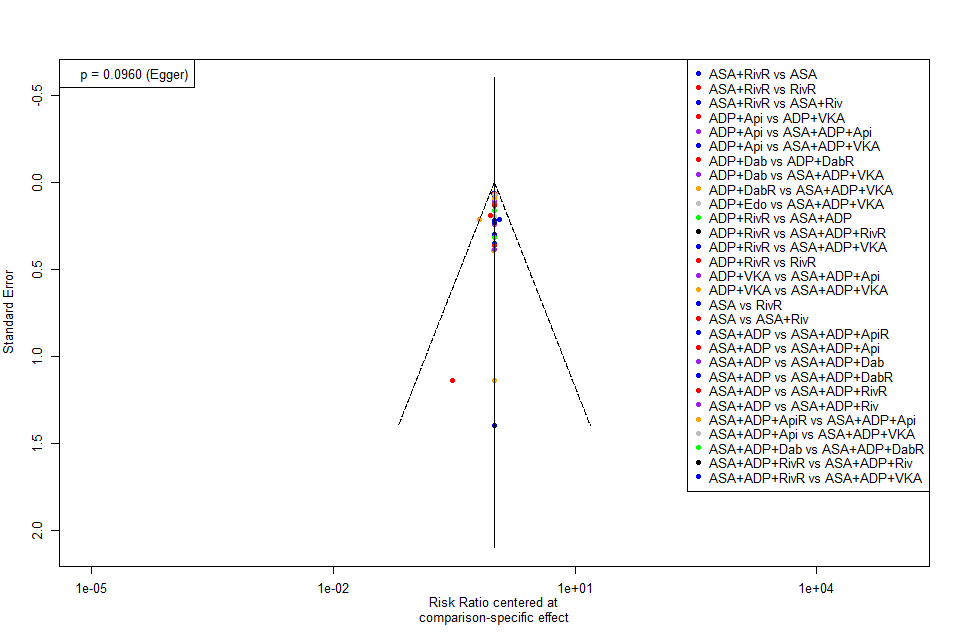


##### B
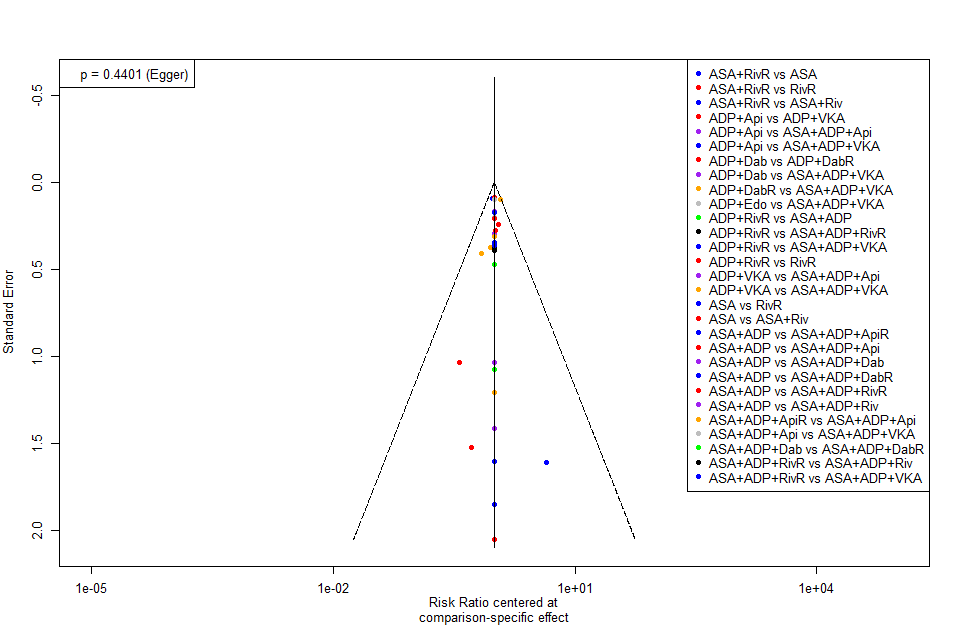


Figure S4 Inconsistency heat plots of MACE outcome (Panel A) and major bleeding (Panel B)
The size of the gray squares indicates the contribution of the direct evidence (shown in the column) to the network evidence (shown in the row). The colors are associated with the change of inconsistency between direct and indirect evidence (shown in the row). Warm colors indicate increase an increase of inconsistency and blue colors indicate a decrease. *Abbreviation*: ASA: aspirin, ADP: P2Y12 ADP receptor antagonist, VKA: Vitamin-K antagonist anticoagulation, Riv: rivaroxaban, Api: apixaban, Dab: dabigatran, Edo: edoxaban, RivR: reduced dose rivaroxaban, ApiR: reduced dose apixaban, DabR: reduced dose dabigatran, vs: versus

**A**
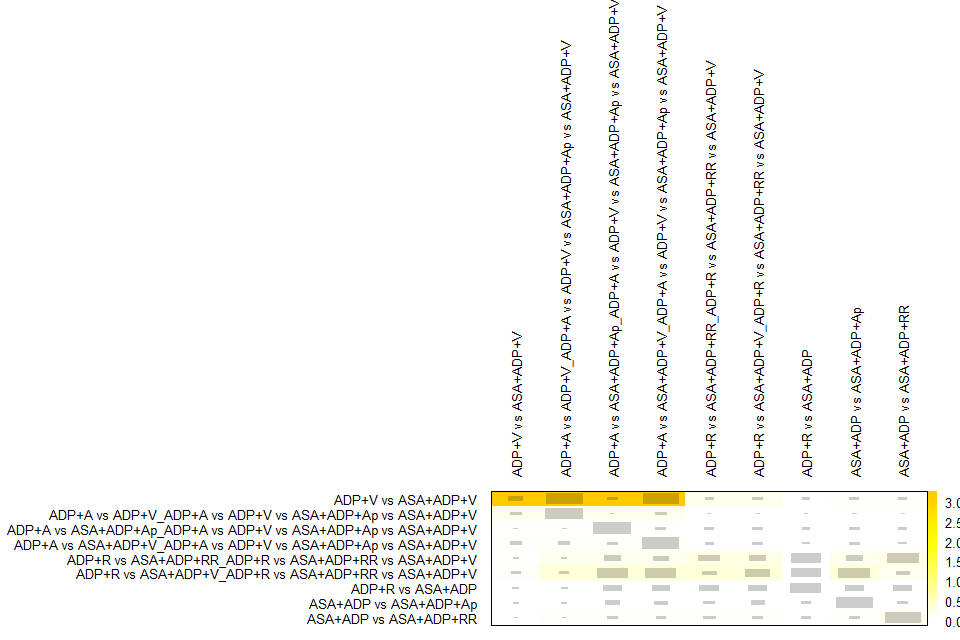


**B**
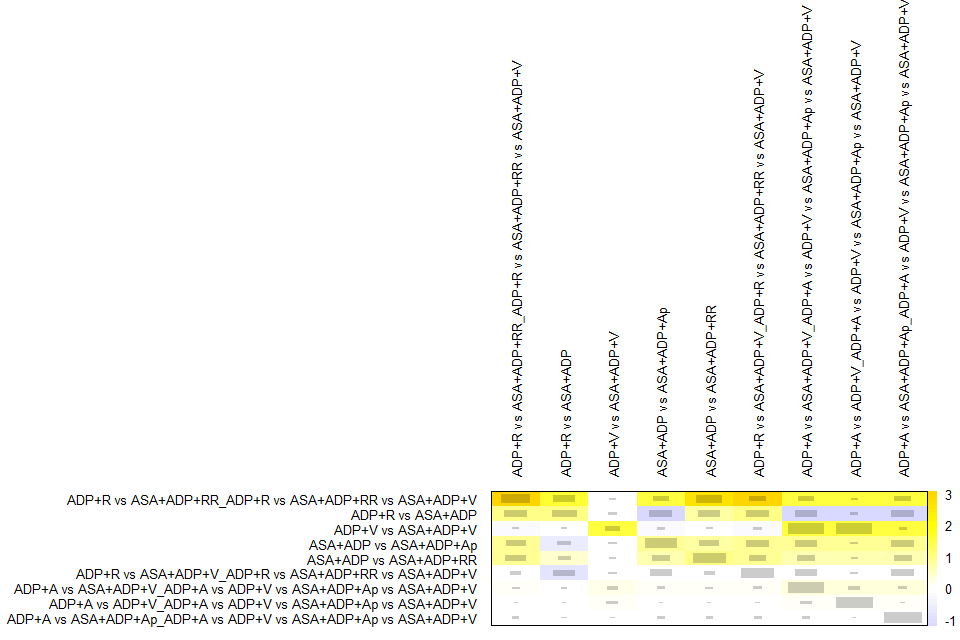


# Figure S5. Net splitting plot of the major cardiovascular adverse events (MACE) (Panel A) and major bleeding (Panel B) showing the consistency between direct and indirect comparisons.

##### Results are presented as risk ratios (RR) and 95% confidence intervals (CI). *Abbreviations*: ASA: aspirin, ADP: P2Y12 ADP receptor antagonist, VKA: Vitamin-K antagonist anticoagulation, Riv: rivaroxaban, Api: apixaban, Dab: dabigatran, Edo: edoxaban, RivR: reduced dose rivaroxaban, ApiR: reduced dose apixaban, DabR: reduced dose dabigatran, vs: versus

##### A
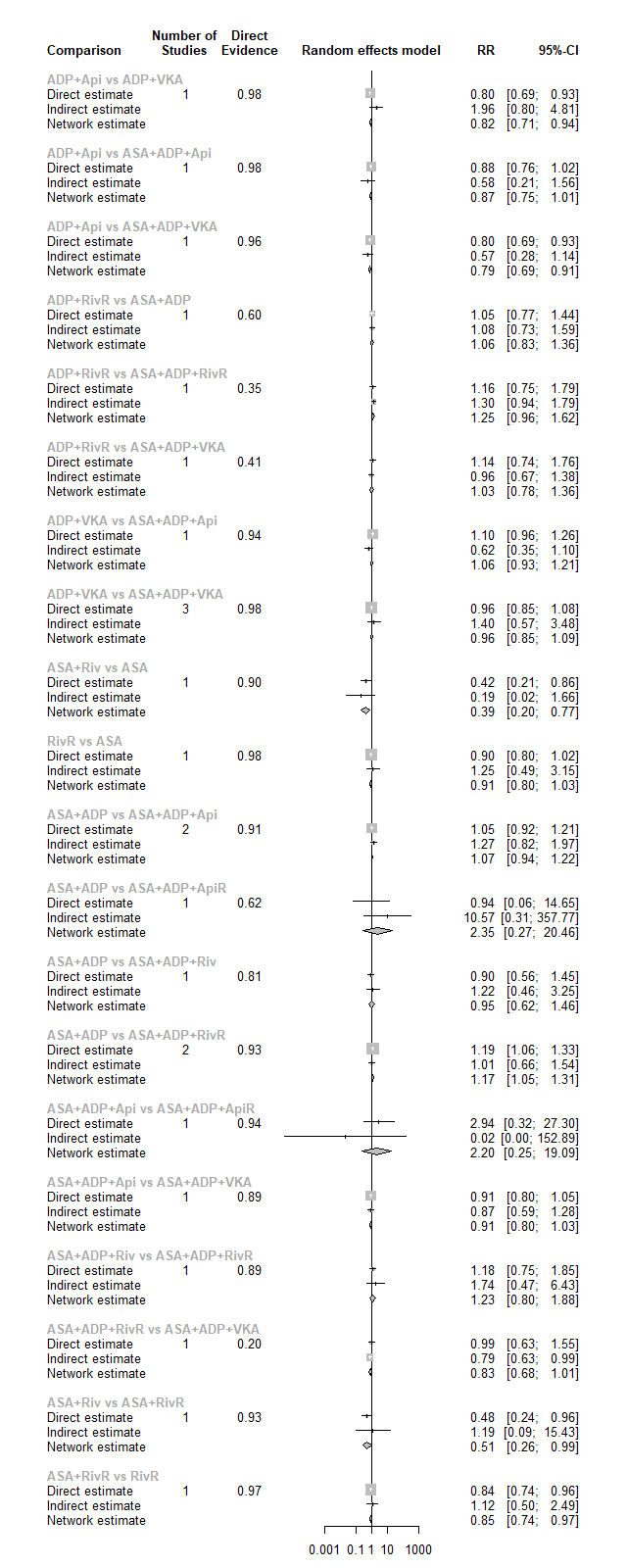
B
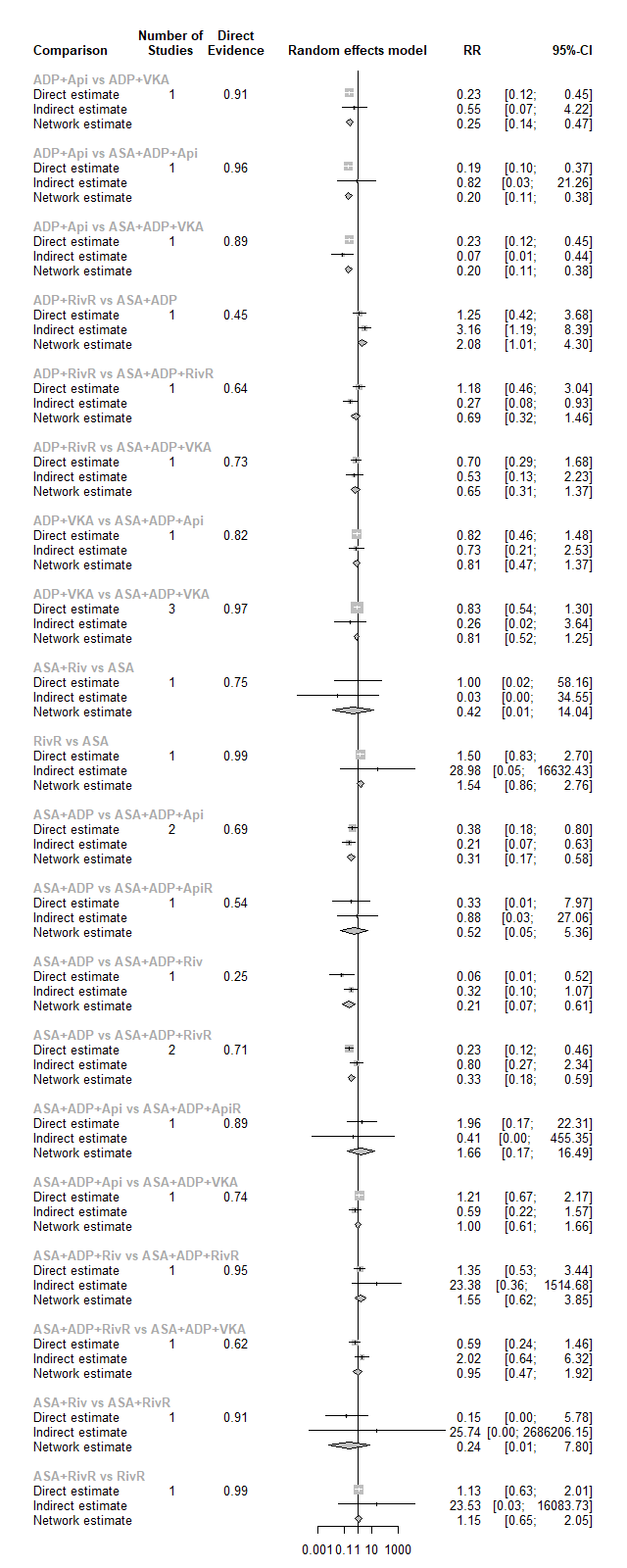


# Figure S6 Results of the network analysis of mortality outcomes.

The forest plots depict the relative risk (RR) and their 95% confidence interval of the overall (Panel A) and the cardiovascular mortality (Panel B) respective to the Vitamin K antagonist (VKA) and double antiplatelet therapy triple regime in the network meta-analysis. Abbreviations: ASA: aspirin, ADP: P2Y12 ADP receptor antagonist, VKA: Vitamin-K antagonist anticoagulation, Riv: rivaroxaban, Api: apixaban, Dab: dabigatran, Edo: edoxaban, RivR: reduced dose rivaroxaban, ApiR: reduced dose apixaban, DabR: reduced dose dabigatran


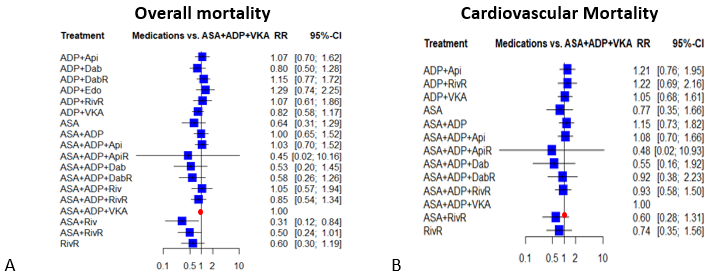


##### Figure S7 Results of the network analysis of ischemic outcomes. The forest plots depict the relative risk (RR) and their 95% confidence interval of the myocardial infarction (Panel A) stroke (Panel B), and the stent thrombosis (Panel C) respective to the Vitamin K antagonist (VKA) and double antiplatelet therapy triple regime in the network meta-analysis. Abbreviations: ASA: aspirin, ADP: P2Y12 ADP receptor antagonist, VKA: Vitamin-K antagonist anticoagulation, Riv: rivaroxaban, Api: apixaban, Dab: dabigatran, Edo: edoxaban, RivR: reduced dose rivaroxaban, ApiR: reduced dose apixaban, DabR: reduced dose dabigatran

#####
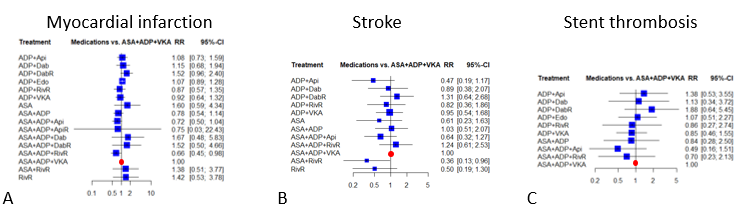


##### Figure S8 Results of the network analysis of minor, and major or minor bleeding events.  The forest plots depict the relative risk (RR) and their 95% confidence interval of the minor bleeding (Panel A) major or cliiniically relevant minor bleeding (Panel B) respective to the Vitamin K antagonist (VKA) and double antiplatelet therapy triple regime in the network meta-analysis. Abbreviations: ASA: aspirin, ADP: P2Y12 ADP receptor antagonist, VKA: Vitamin-K antagonist anticoagulation, Riv: rivaroxaban, Api: apixaban, Dab: dabigatran, Edo: edoxaban, RivR: reduced dose rivaroxaban, ApiR: reduced dose apixaban, DabR: reduced dose dabigatran


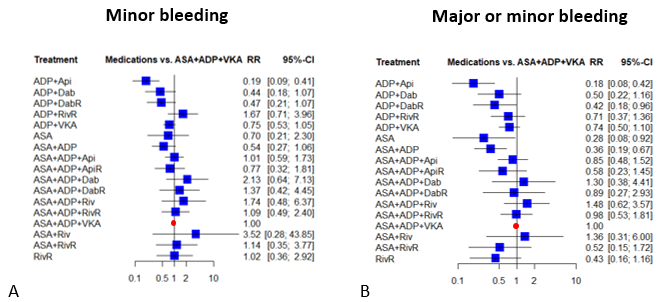


# Table S1 Baseline characteristics of the involved studies

Abbreviations: ACS: acute coronary syndrome, ADP: adenosine diphosphate, ASA: aspirin, BARC: Bleeding Academic Research Consortium, CVD: cardiovascular death, early discont: early discontinuation, GUSTO: Global Utilization Of Streptokinase And Tpa For Occluded Arteries, ISTH: International Society on Thrombosis and Haemostasis, mo: month, MI: myocardial infarction, no: number of, RCT: randomized controlled trial, Riv: rivaroxaban, ST: stent thrombosis, TIMI: Thrombolysis in Myocardial Infarction, UA: unstable angina

| **Study** | **Author**  **(year)** | **Country** | **Study design, Phase** | **No. of patients** | **Treatment** | **Follow-up (mean) (mo), Early discont. (+/-)** | **Primary endpoint** | **Secondary endpoint** | **Bleeding definition** |
| --- | --- | --- | --- | --- | --- | --- | --- | --- | --- |
| ATLAS ACS-TIMI 46 | Mega et al.  (2009) | worldwide | RCT, multicenter, 2 by 2 factorial, 2 | 3491 | Stratum 1: ASA + Rivaroxaban / Riv (reduced) Stratum 2: ASA +ADP + Riv / Riv (reduced) | 6, - | Mortality, MI, stroke, revascularization | Mortality, MI, stroke | TIMI |
| APPRAISE-2 | Alexander et al. (2011) | worldwide | RCT, multicenter, 3 | 7392 | ASA +ADP +/- Apixaban | 15, + | Composite of CVD, MI, ischemic stroke; major bleeding | Composite of: CVD, MI, stroke, UA; ST | ISTH, GUSTO, TIMI |
| ATLAS ACS 2-TIMI 51 | Mega et al. (2011) | worldwide | RCT, multicenter,3 | 15526 | ASA + ADP +/- Rivaroxaban (reduced) | 13, - | Composite of CVD, MI, stroke | Mortality, MI, stroke, ST | TIMI |
| REDEEM | Oldgren et al. (2011) | worldwide | RCT, multicenter, 2 | 1861 | ASA + ADP +/- Dabigatran /Dabi (reduced) | 6, - | Major and minor bleeding | Reduction in D-dimer levels, Composite of CVD, non-fatal MI, non-haemorrhaic stroke; mortality | ISTH,  TIMI, GUSTO |
| APPRAISE-J | Ogawa et al. (2013) | Japan | RCT, multicenter, 2 | 150 | ASA + ADP + Apixaban/Apixaban (reduced) | 6, + | Composite of major or clinically relevant bleeding | Major and minor bleeding, mortality, nonfatal MI, UA, stroke | ISTH |
| WOEST | Dewilde et al. (2013) | Belgium-Netherland | RCT, multicenter,4 | 573 | VKA + Clopidogrel +/- ASA | 12, - | Major and minor bleeding | Composite of: MI, stroke, TVR, ST | TIMI, GUSTO, BARC |
| ISAR-TRIPLE | Fielder et al. (2015) | Germany | RCT, multicenter, 4 | 614 | ASA + ADP +/- VKA | 9, - | Composite of death, MI, ST, stroke, major bleeding | Cumulative incidence of CVD, MI, ST, ischemic stroke; major and minor bleeding | TIMI, BARC |
| PIONEER | Gibson et al. (2016) | worldwide | RCT, multicenter, 2 by 2 factorial, 3 | 4614 | VKA/Apixaban + ADP +/- ASA | 6, - | Composite of major and minor bleeding | Composite of ischemic events: CVD, ST, MI, stroke, urgent revascularization, CV hospitalization | ISTH |
| COMPASS | Eikelboom et al (2017) | worldwide | RCT, multicenter, 3 | 27395 | ASA/Rivaroxaban (reduced) +/- ASA/Rixaroxaban (reduced) | 23, + | Composite of CVD, stroke, MI; major bleeding | Composite of ischemic stroke, MI, acute limb ischemia, CVD; mortality | ISTH |
| GEMINI-ACS-1 | Ohman et al. (2017) | worldwide | RCT, multicenter, 2 | 3037 | ADP + ASA/Rivaroxaban (reduced) | 11, - | Major and minor bleeding | Composite of: CVD, MI, stroke, ST; mortality | TIMI, GUSTO, BARC, ISTH |
| RE-DUAL PCI | Cannon et al. (2017) | worldwide | RCT, multicenter, 3 | 2725 | VKA/Dabigatran + ADP +/- ASA | 14, + | Major and minor bleeding | Composite of: MI, stroke, systolic embolism; death, unplanned TVR | ISTH,  TIMI |
| AFIRE | Yasuda et al. (2019) | Japan | RCT, multicenter, 3 | 2236 | Rivaroxaban (reduced) +/- ADP | 24,1, + | Composite of stroke, systemic embolism, MI, UA, mortality; major bleeding | Individual components of the primary endpoint; TIA, systemic arterial embolism, venous thromboembolism, revascularization, ST | ISTH |
| AUGUSTUS | Lopes et al. (2019) | worldwide | RCT, multicenter, 2 by 2 factorial, 2 | 4614 | VKA/Apixaban + ADP +/- ASA | 6, - | Composite of major and minor bleeding | Composite of ischemic events (CVD, ST, MI, stroke, urgent revascularization, CV hospitalization) | ISTH |
| ENTRUST | Vranckx et al. (2019) | worldwide | RCT, multicenter, 3b | 1506 | ADP +/- ASA+ VKA/Edoxaban | 12, - | Composite of CVD, stroke, systemic embolic events (SEE), MI, ST | Net clinical benefit, major and minor bleeding | ISTH,  TIMI, BARC |
| MANJUSRI | Lu et al 2021 | China | RCT, multicentre, 3 | 296 | ADP +/- ASA + VKA |  | Composite of major and minor bleeding | Composite of ischemic events | TIMI |
